# Supplementary material for: Non-Faradaic optoelectrodes for safe electrical neuromodulation
Source: Nat Commun. 2024 Jan 9;15:405. doi: 10.1038/s41467-023-44635-8 (PMC10776784; doi:10.1038/s41467-023-44635-8)
Supplement: Supplementary file 1 — Supplementary information [file 41467_2023_44635_MOESM1_ESM.pdf]

Supplementary Information for

Non-Faradaic Optoelectrodes for Safe Electrical Neuromodulation

Jian Chen[1#], Yanyan Liu[1,2#], Feixiang Chen[1#], Mengnan Guo[1], Jiajia Zhou[3], Pengfei Fu[2], Xin Zhang[2], Xueli Wang[4], He Wang[5], Wei Hua[2], Jinquan Chen[4], Jin Hu[2], Ying Mao*[2], Dayong Jin*[3,6], and Wenbo Bu*[1,2]

[1]Department of Materials Science, State Key Laboratory of Molecular Engineering of Polymers, Fudan University, Shanghai 200433, China

[2]Department of Neurosurgery, Huashan Hospital, Fudan University, Shanghai 200041, China

[3]Institute for Biomedical Materials and Devices (IBMD), Faculty of Science, University of Technology Sydney, Sydney, New South Wales 2007, Australia

[4]Sate Key Laboratory of Precision Spectroscopy, East China Normal University, Shanghai 200062, China

[5]Institute of Science and Technology for Brain Inspired Intelligence, Fudan University, Shanghai 200433, China

[6]Eastern Institute for Advanced Study, Eastern Institute of Technology, Ningbo, Zhejiang, 315200, P.R. China

[#]These authors contributed equally

*Corresponding authors, maoying@fudan.edu.cn; dayong.Jin@uts.edu.au; wbbu@fudan.edu.cn

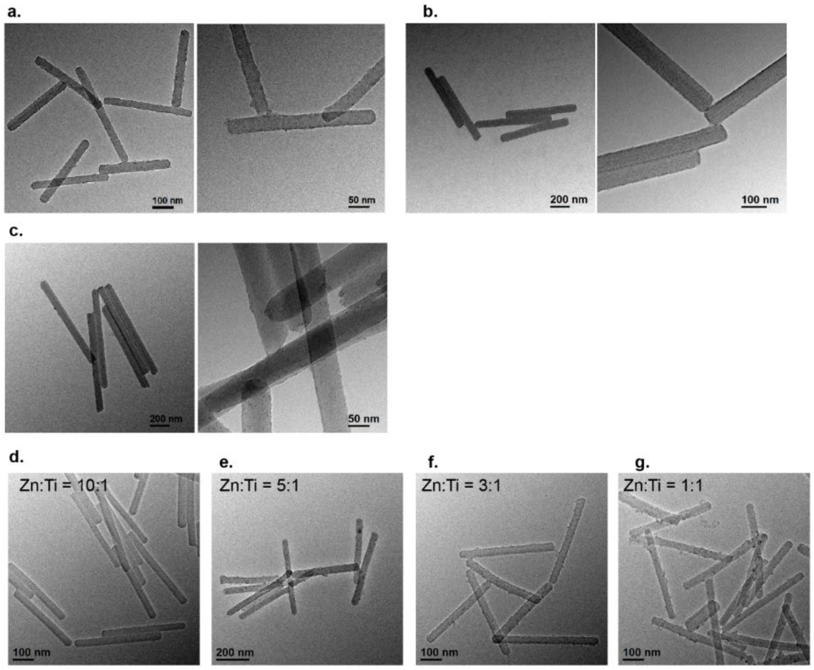

Supplementary Figure S1 | a-c, TEM images of ZST nanorods with different sizes. **d-g**, TEM images of ZST nanorods with varied TiO_2 ratios.

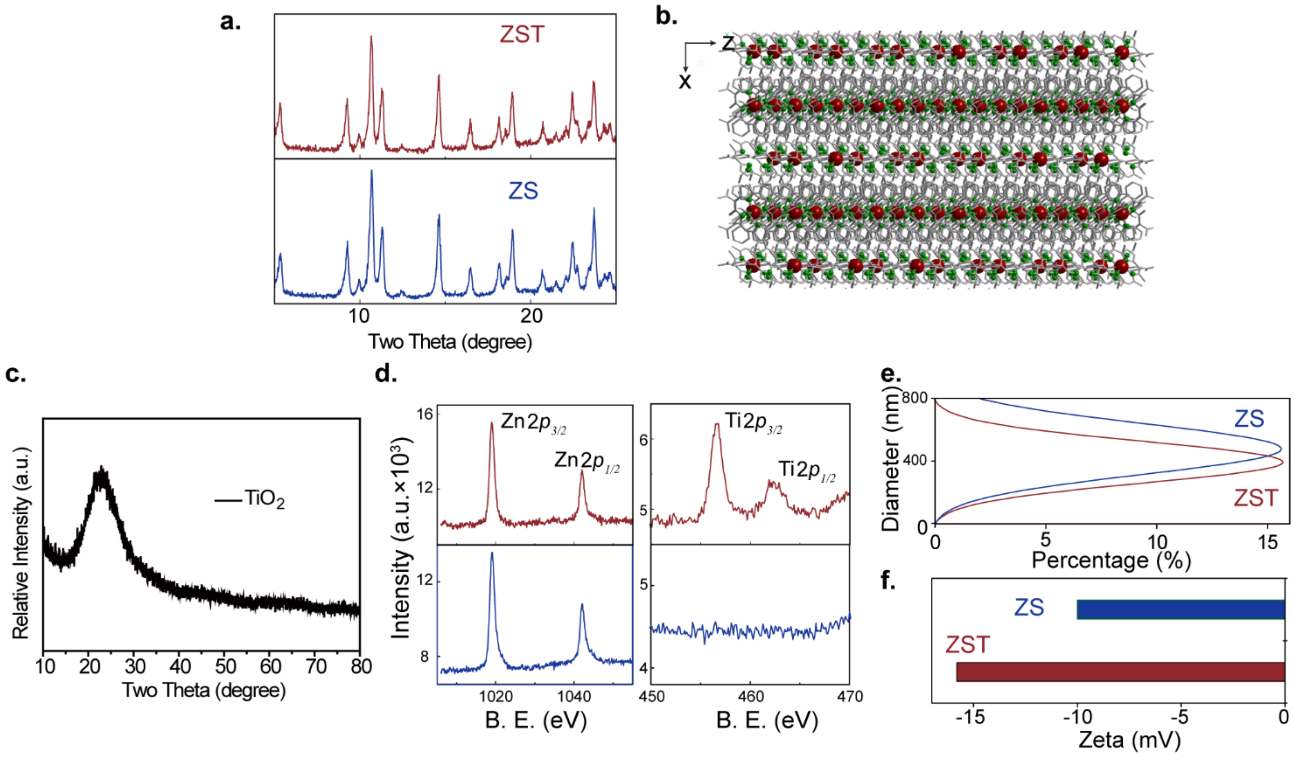

Supplementary Figure S2 | a, XRD spectra of ZS and ZST, and **b,** the corresponding simulated crystal structure of ZS (plan-view, 270779.cif). Zn (red) and N (green). **c,** XRD spectrum of amorphous TiO_2. **d,** XPS spectra of ZS and ZST in different binding-energy ranges. **e-f,** DLS sizes and zeta potentials of ZS and ZST.

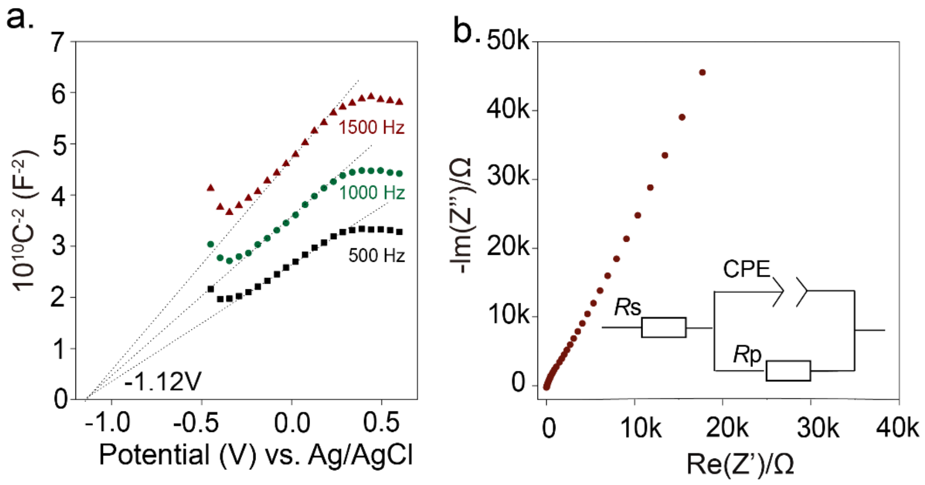

Supplementary Figure S3 | a, Mott-Schottky analysis of ZS. **b**, Electrochemical impedance spectrum of ZST with the insert equivalent circuit model.

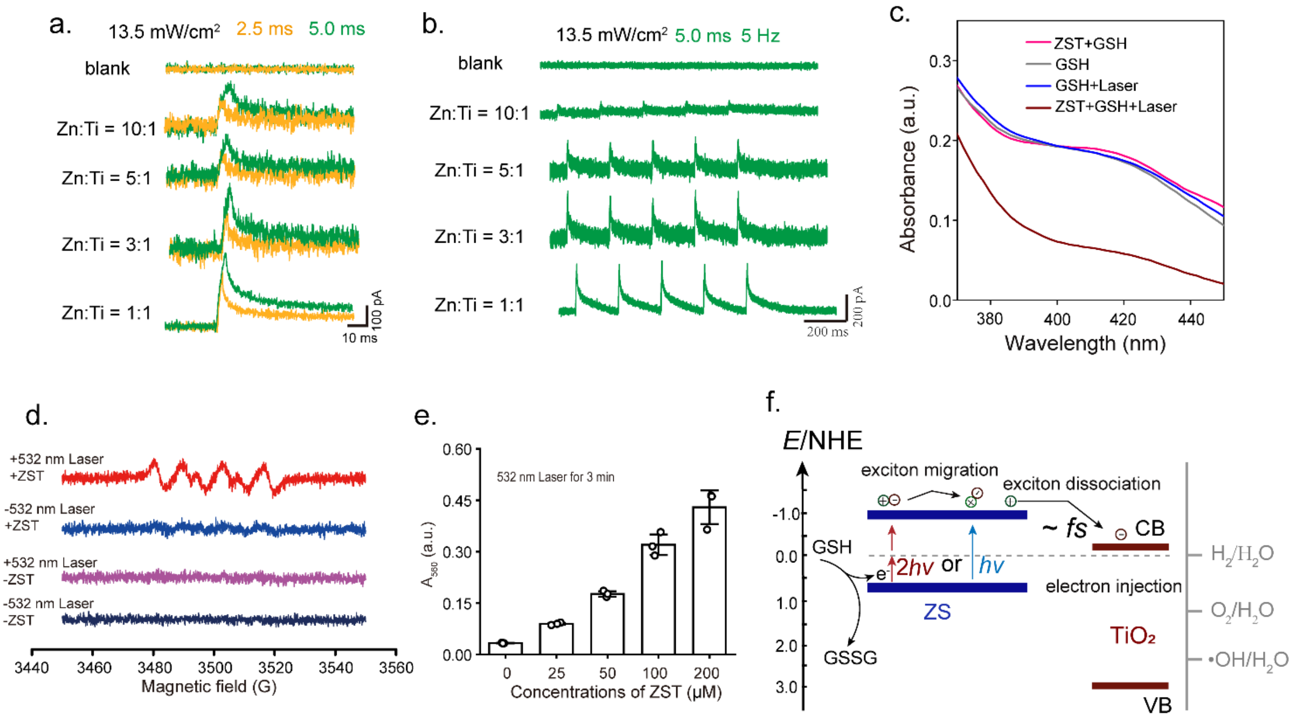

Supplementary Figure S4 | a, Photocurrent of ZST excited by 532 nm laser at varied light powers and light irradiation times. **b,** The pulsed photocurrent acquired by ZST with varied Zn/Ti ratios under pulsed light stimulation. **c,** The absorption of GSH in solutions with different treatments. A fast consumption of GSH was detected in ZST solution after 532 nm laser irradiation, due to its supply of electrons for significant dye regeneration. **d,** EPR spectra in solution with or without ZST, and with or without laser irradiation. DMPO was used as radical scavenger. **e,** Superoxide anion kit used for quantitative detection of $O_2^{\cdot-}$ with varied ZST concentrations after 532 nm laser irradiation for 3 min. **f,** The process of photon induced consecutive charge transfer reactions in ZST and the final formed redox potential. The whole photo-stimulation process of ZST is

capacitive in photocapacitive mode, without water hydrolysis or ROS generation, due to the insufficient photo-electrochemical potential of ZST, beneficial for its long-term neurostimulation.

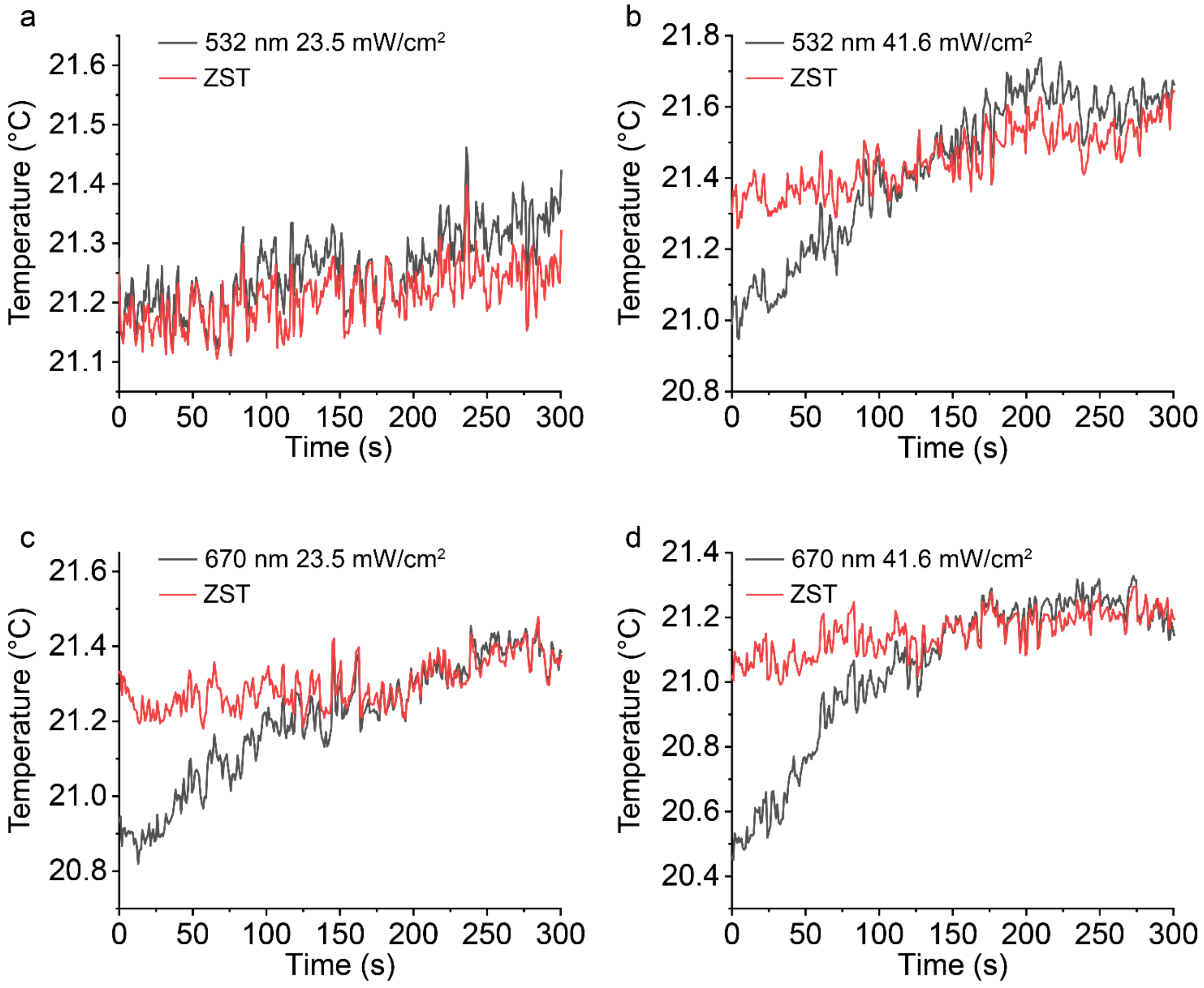

Supplementary Figure S5 | The temperature changes over time for ZST under laser illumination at a wavelength of 532 nm with the power of (a) 23.5 and (b) 41.6 mW/cm^2**,** 670·nm with the power of (c) 23.5 and (d) 41.6 mW/cm^2.

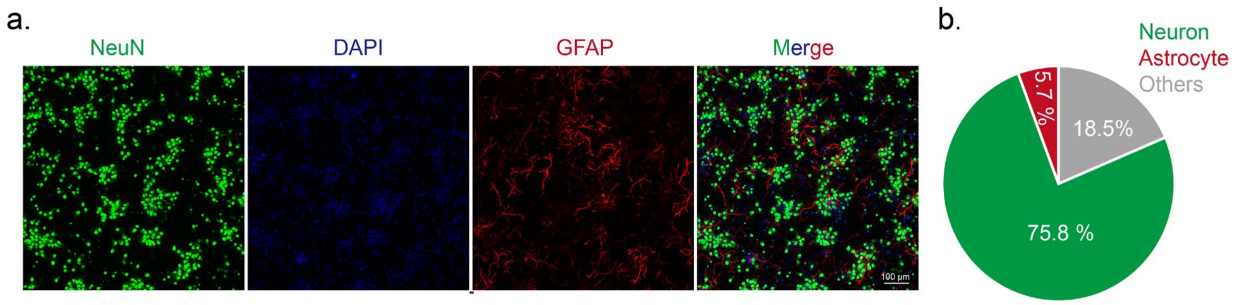

Supplementary Figure S6 | a Purity of primary neuron cultures as evidenced by immunolabeling with NeuN (neuronal marker), GFAP (astrocyte marker) and DAPI (cellular nucleus marker). **b,** The average percentage

of various cell types in primary cell cultures varies (neurons, 75.8% ± 2.4%; astrocyte, 5.7% ± 0.3%; others, 18.5% ± 2.3%; n = 8 plates of cells).

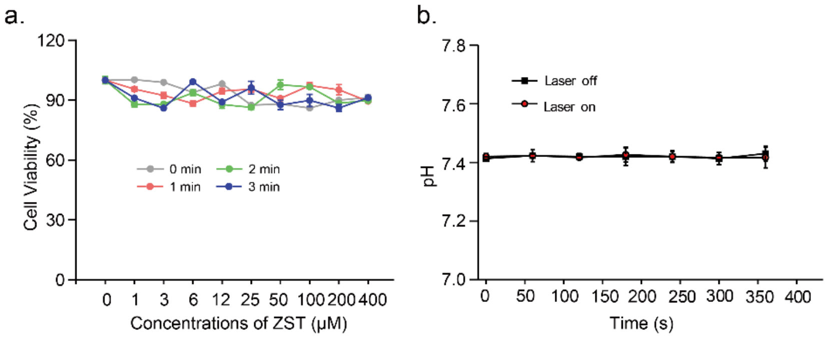

Supplementary Figure S7 | a, The viability of cells with varied ZST concentrations and 532 nm laser irradiation times by a typical MTT assay. **b**, The change of pH in culture after 532 nm laser irradiation for different times.

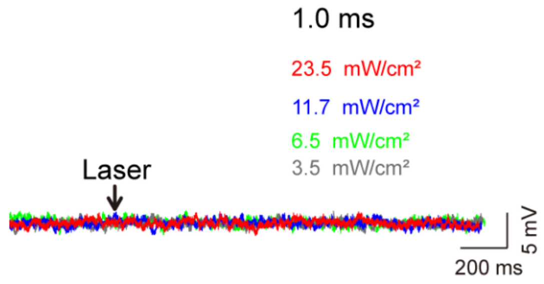

Supplementary Figure S8 | The trace of AP in neurons pre-treated by ZS with different 532 nm laser powers.

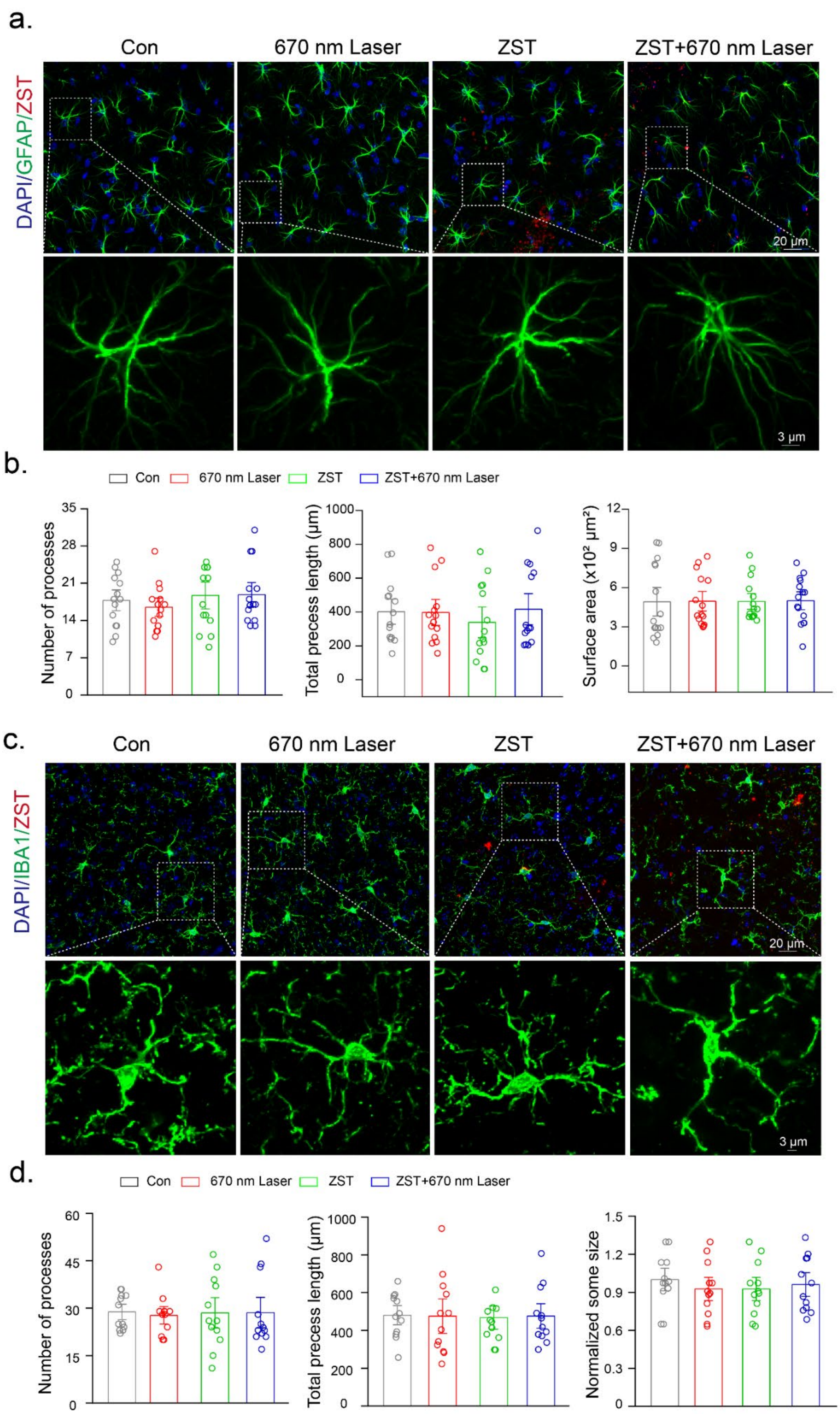

Supplementary Figure S9 | a, The immunostaining of GFAP expressed in mice M2 that was collected 30 days after different treatments. **b**, The corresponding statistical numbers of process, total process length,

and surface area of astrocytes. **c,** The immunostaining of IBA1 expressed in mice M2 that was collected 30 days after different treatments. **d,** The corresponding statistical numbers of process, total process length, and cell body size of microglia. The results are shown as mean ± sem by two way ANOVA analysis followed by Bonferroni's post hoc test,(n = 14 cells in astrocytes; n = 12 cells in microglia; N = 3 mice in each group (*p < 0.05, **p < 0.01, ***p < 0.001)).

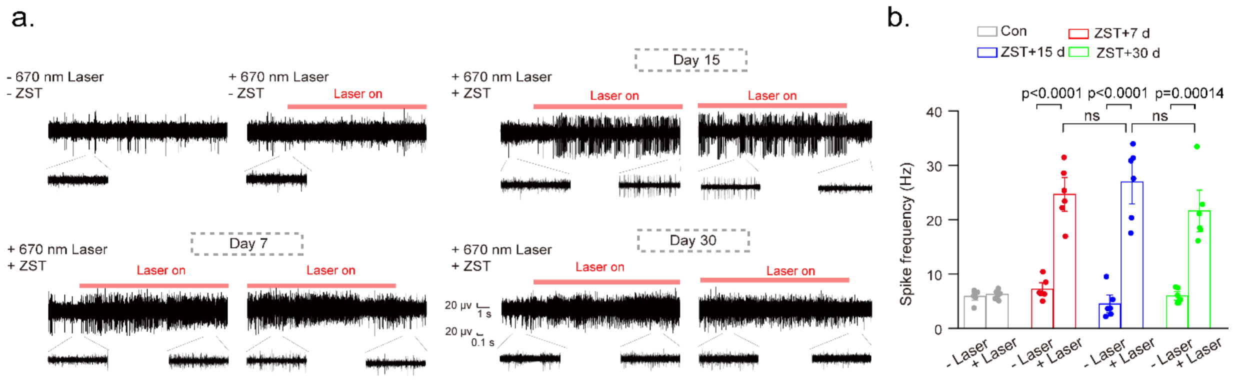

Supplementary Figure S10 | a, *In vivo* multichannel electrophysiology used to monitor the neuronal firing of mice under different treatments on the 7th, 15th, and 30th days after ZST was injected in M2. (-Laser: no laser; +Laser: laser irradiation; -ZST: no ZST; +ZST). **b,** The results are shown as mean ± SD by The two-way repeated measures analysis of variance (ANOVA) by Bonferroni's post hoc test, N=6 neurons from 3 mice in each group.
