# Supplementary material for: Non-Faradaic optoelectrodes for safe electrical neuromodulation
Source: Nat Commun. 2024 Jan 9;15:405. doi: 10.1038/s41467-023-44635-8 (PMC10776784; doi:10.1038/s41467-023-44635-8)
Supplement: Supplementary file 3 — Description of Additional Supplementary Files [file 41467_2023_44635_MOESM3_ESM.pdf]

Description of Additional Supplementary Files

Supplementary Movies

Supplementary Movie 1. The video of hind limb movement at 670 nm laser stimulation (it is a real time recording).

Supplementary Movie 2. The video of hind limb movement at 850 nm laser stimulation (it is a real time recording).

Supplementary Datasets

Supplementary dataset 1 . The treatment protocol for Parkinson's disease.

Supplementary dataset 2. The comparison of different electrodes used in neural stimulation.

Supplementary dataset 3 . The comparison of nanoscale optoelectrodes with optogenetics and chemogenetic
