# Supplementary material for: Non-Faradaic optoelectrodes for safe electrical neuromodulation
Source: Nat Commun. 2024 Jan 9;15:405. doi: 10.1038/s41467-023-44635-8 (PMC10776784; doi:10.1038/s41467-023-44635-8)
Supplement: Supplementary file 7 — Reporting Summary [file 41467_2023_44635_MOESM7_ESM.pdf]

nature portfolio

Corresponding author(s): Ying Mao, Dayong Jin, Wenbo Bu

Last updated by author(s): Dec 5, 2023

# Reporting Summary

Nature Portfolio wishes to improve the reproducibility of the work that we publish. This form provides structure for consistency and transparency in reporting. For further information on Nature Portfolio policies, see our Editorial Policies and the Editorial Policy Checklist.

## Statistics

For all statistical analyses, confirm that the following items are present in the figure legend, table legend, main text, or Methods section.

| n/a | Confirmed | |
|---|---|---|
| ☐ | ☒ | The exact sample size ($n$) for each experimental group/condition, given as a discrete number and unit of measurement |
| ☐ | ☒ | A statement on whether measurements were taken from distinct samples or whether the same sample was measured repeatedly |
| ☐ | ☒ | The statistical test(s) used AND whether they are one- or two-sided<br>*Only common tests should be described solely by name; describe more complex techniques in the Methods section.* |
| ☐ | ☒ | A description of all covariates tested |
| ☐ | ☒ | A description of any assumptions or corrections, such as tests of normality and adjustment for multiple comparisons |
| ☐ | ☒ | A full description of the statistical parameters including central tendency (e.g. means) or other basic estimates (e.g. regression coefficient) AND variation (e.g. standard deviation) or associated estimates of uncertainty (e.g. confidence intervals) |
| ☐ | ☒ | For null hypothesis testing, the test statistic (e.g. $F$, $t$, $r$) with confidence intervals, effect sizes, degrees of freedom and $P$ value noted<br>*Give P values as exact values whenever suitable.* |
| ☒ | ☐ | For Bayesian analysis, information on the choice of priors and Markov chain Monte Carlo settings |
| ☐ | ☒ | For hierarchical and complex designs, identification of the appropriate level for tests and full reporting of outcomes |
| ☒ | ☐ | Estimates of effect sizes (e.g. Cohen's $d$, Pearson's $r$), indicating how they were calculated |

*Our web collection on statistics for biologists contains articles on many of the points above.*

## Software and code

Policy information about availability of computer code

| | |
|---|---|
| Data collection | Nikon A1 software 5.21.03, catwalk XT 10.6, patchclampfit 10.5, Visu track, SlideViewer |
| Data analysis | Jade 6.5, OriginPro 9.1, Clampfit 10.5 and GraphPad Prism 8.0.2 were used for plotting. |

For manuscripts utilizing custom algorithms or software that are central to the research but not yet described in published literature, software must be made available to editors and reviewers. We strongly encourage code deposition in a community repository (e.g. GitHub). See the Nature Portfolio guidelines for submitting code & software for further information.

## Data

Policy information about availability of data

All manuscripts must include a data availability statement. This statement should provide the following information, where applicable:

- Accession codes, unique identifiers, or web links for publicly available datasets
- A description of any restrictions on data availability
- For clinical datasets or third party data, please ensure that the statement adheres to our policy

The authors declare that the main data supporting the results in this study are available within the paper and its Supplementary Information. Additional datasets are available from the corresponding author on reasonable request.

# Field-specific reporting

Please select the one below that is the best fit for your research. If you are not sure, read the appropriate sections before making your selection.

☒ Life sciences ☐ Behavioural & social sciences ☐ Ecological, evolutionary & environmental sciences

For a reference copy of the document with all sections, see nature.com/documents/nr-reporting-summary-flat.pdf

# Life sciences study design

All studies must disclose on these points even when the disclosure is negative.

| | |
|---|---|
| Sample size | Sample sizes were determined on the basis of prior experimental work or a pilot study. No statistical calculations were made to determine sample size. |
| Data exclusions | No data were excluded. |
| Replication | All experiments were carried out with at least 3 replicate samples for each experimental group. All data are representative of at least three independent experiments with similar results, which was described in corresponding figure legends. |
| Randomization | Samples were allocated into experimental groups at random. |
| Blinding | The investigator was blinded to the group allocation during data collection such as in Behavior test, Westernblot, Immuno-fluorescences, H&E staining. For other experiments,blinding was not possible since the primary investigators performed the experiments from the beginning to the end due to the technical nature of the experiments. |

# Reporting for specific materials, systems and methods

We require information from authors about some types of materials, experimental systems and methods used in many studies. Here, indicate whether each material, system or method listed is relevant to your study. If you are not sure if a list item applies to your research, read the appropriate section before selecting a response.

## Materials & experimental systems

| n/a | Involved in the study | |
|---|---|---|
| ☐ | ☒ | Antibodies |
| ☒ | ☐ | Eukaryotic cell lines |
| ☒ | ☐ | Palaeontology and archaeology |
| ☐ | ☒ | Animals and other organisms |
| ☒ | ☐ | Human research participants |
| ☒ | ☐ | Clinical data |
| ☒ | ☐ | Dual use research of concern |

## Methods

| n/a | Involved in the study | |
|---|---|---|
| ☒ | ☐ | ChIP-seq |
| ☒ | ☐ | Flow cytometry |
| ☒ | ☐ | MRI-based neuroimaging |

## Antibodies

**Antibodies used**

1. anti-β-actin antibody (Abcam, ab8227, 1:1000),
2. anti-c-Fos antibody (Abcam, ab214672, 1:500),
3. anti-Tyrosine Hydroxylase antibody (Abcam, ab6211, 1:500)
4. anti-Tyrosine Hydroxylase antibody (Abcam, ab6211, 1:500),
5. anti-GFAP antibody (Abcam, ab68428, 1:500),
6. anti-IBA1 antibody (Wako, 019-19741, 1:500),
7. anti-NeuN antibody (Abcam, ab236869, 1:500)
8. anti-GFAP antibody (CST, 3670S, 1:500)

**Validation**

1. anti-β-actin antibody (Abcam, ab8227, 1:1000), https://www.abcam.cn/products/primary-antibodies/beta-actin-antibody-ab8227.html
2. anti-c-Fos antibody (Abcam, ab214672, 1:500), https://www.abcam.cn/products/primary-antibodies/c-fos-antibody-epr20769-ab214672.html
3. anti-Tyrosine Hydroxylase antibody (Abcam, ab6211, 1:500),https://www.abcam.cn/products/primary-antibodies/tyrosine-hydroxylase-antibody-ab6211.html
4. anti-Tyrosine Hydroxylase antibody (Abcam, ab6211, 1:500), https://www.abcam.cn/products/primary-antibodies/tyrosine-hydroxylase-antibody-ab6211.html
5. anti-GFAP antibody (Abcam, ab68428, 1:500), https://www.abcam.cn/products/primary-antibodies/gfap-antibody-epr1034y-ab68428.html
6. anti-IBA1 antibody (Wako, 019-19741, 1:500), https://labchem-wako.fujifilm.com/asia/product/result/product.html?fw=019-19741

7. anti-NeuN antibody (Abcam, ab236869, 1:500), https://www.abcam.cn/products/primary-antibodies/neun-antibody-epr21902-ab236869.html
8. anti-GFAP antibody (CST, 3670S, 1:500), https://www.cellsignal.cn/products/primary-antibodies/gfap-ga5-mouse-mab/3670

## Animals and other organisms

Policy information about studies involving animals; ARRIVE guidelines recommended for reporting animal research

| | |
|---|---|
| Laboratory animals | C57BL/6J male mice (8–10 weeks) and 16-day Shjh:SD pregnant rats |
| Wild animals | The study did not involve wild animals. |
| Field-collected samples | The study did not involve samples collected from the field. |
| Ethics oversight | All animal experiments were conducted according to the guidelines of the institutional animal care and use committee and the experiments animal ethics committee of east normal university, and the accreditation number was m+R20190701. |

Note that full information on the approval of the study protocol must also be provided in the manuscript.
